# Association of the serum microRNA-29 family with cognitive impairment in Parkinson's disease

**DOI:** 10.18632/aging.103458

**Published:** 2020-07-09

**Authors:** Linlin Han, Yilin Tang, Xiaochen Bai, Xiaoniu Liang, Yun Fan, Yan Shen, Fang Huang, Jian Wang

**Affiliations:** 1 Department of Neurology and National Clinical Research Center for Aging and Medicine, Huashan Hospital, Fudan University, Shanghai 200040, China; 2 State Key Laboratory of Medical Neurobiology and MOE Frontiers Center for Brain Science, Institutes of Brain Science, Fudan University, Shanghai 200032, China

**Keywords:** Parkinson’s disease, mild cognitive impairment, dementia, microRNA

## Abstract

We aimed to examine whether miRNA-29s (miR-29s) in serum are associated with cognitive impairment in Parkinson’s disease (PD). Thirty-nine PD patients with normal cognition (PD-NC), 37 PD patients with mild cognitive impairment (PD-MCI), 22 PD patients with dementia (PDD) and 40 healthy controls were recruited. Detailed clinical evaluations and a schedule of neuropsychological tests were administered to all patients. MiR-29s expression in serum samples was assessed using reverse-transcription quantitative real-time PCR. We found that the levels of all three miR-29s in the PDD group were significantly lower than those in the PD-NC group (p < 0.05). In addition, the miR-29b level was downregulated in the PD-MCI group with respect to that in the PD-NC group (p < 0.05). After adjusting for years of education and the UPDRS-III subscore using a multivariate model, miR-29s showed significant associations with PDD. MiR-29b levels were shown to be associated with different subsets of PD cognition and could accurately discriminate PDD from non-PDD (area under the curve (AUC) = 0.859; 95% CI, 0.7817-0.9372). Further analysis of the cognitive domains found that the miR-29s levels were all associated with memory performance in PD patients. In summary, miR-29s are associated with cognitive impairment in PD.

## INTRODUCTION

Cognitive impairment (CI) is the most prevalent and debilitating nonmotor symptom in Parkinson’s disease (PD) [1], ranging from mild cognitive impairment (PD-MCI) to dementia (PDD). However, the current diagnosis of PDD, based on both the DSM-IV criteria and the proposed clinical diagnostic criteria, is time-consuming and somewhat subjective [2]. Reliable and easily applicable biomarkers for PDD or even earlier stages of impairment, such as PD-MCI, are urgently needed.

MicroRNAs (miRNAs) are single-stranded sets of 21-22 nucleotides that act as posttranscriptional modulators by inhibiting or promoting the degradation of their mRNA targets [3, 4]. The potential of miRNAs as peripheral biomarkers of dementia, such as Alzheimer’s disease (AD) [5], vascular dementia [6] and frontotemporal dementia (FTD) [7], is being actively pursued and shows promise. It has been reported that miRNAs contribute to cognitive impairment by playing critical roles in neurodevelopment, synaptic plasticity, memory and the regulation of neurodegenerative disease-associated pathological proteins [8, 9]. However, to our knowledge, no study has previously investigated the roles of miRNAs in cognitive impairment in PD.

We previously reported that the members of the miR-29 family (miR-29a, miR-29b and miR-29c), a group of brain-specific miRNAs, were downregulated in the serum of PD patients and showed a decreasing trend related to more severe Parkinsonism [10]. Considering that cognitive performance worsens with increasing disease severity in PD [11] and the critical role of miR-29s in neuronal survival [12], aging [13], and synaptic plasticity [14], we wondered whether miR-29s were associated with cognitive deficits in PD. To explore this, we compared serum miR-29s expression in PD patients with different cognitive states and evaluated their potential diagnostic accuracy for PDD and PD-MCI patients.

## RESULTS

### Clinical characteristics and neuropsychological tests of participants

We enrolled 98 PD patients and 40 healthy controls (HCs) in total. The patients were classified into 3 groups: patients with PDD (n = 22), patients with PD-MCI (n = 37), and patients with PD-NC (n = 39). The four groups were gender- and age-matched (p > 0.05; Table 1). No group differences were found with respect to ESS scores, SSST-12 scores or RBDSQ scores among the PD patients (all p > 0.05). The PDD group, followed by the PD-MCI group, had the longest disease duration, the most sever motor impairment (H&Y, UPDRS-III), and the highest GDS scores and hallucination incidence, while having the shortest years of education. The LEDs in the PDD and PD-MCI groups were both higher than those in the PD-NC group.

**Table 1:** Clinical characteristics of patients with Parkinson’s disease and healthy controls.

	**HC (n=40)**	**PD-NC (n=39)**	**PD-MCI (n=37)**	**PDD (n=22)**	**p**value**
Age, years	63.75(5.611)	61.49(5.529)	61.43(6.842)	61.86(6.657)	0.0.304
Gender, men*	23(57.5%)	22(56.4%)	26(70.3%)	11(50%)	0.427
Education, years	/	12.28(3.112)	8.34(4.771)	9.55(4.361)	<0.001
Disease duration, months	/	32.3(29.008)	63.82(61.371)	82.17(62.377)	<0.01
UPDRS-III subscore^# ^	/	26.18(16.153)	34.03(13.889)	39.63(17.839)	<0.01
Hoehn and Yahr stage**	/	1.89(0.924)	2.46(1.016)	2.88(0.947)	<0.01
ESS score	/	6.21(5.095)	7.26(5.249)	7.19(4.976)	0.631
GDS score	/	10.79(6.799)	11.26(6.934)	15.71(6.001)	<0.05
SSST 12 score	/	5.18(2.522)	4.64(2.332)	3.6(2.479)	0.068
RBDSQ score	/	4.32(3.154)	5.26(3.579)	5.95(3.203)	0.193
Hallucination*	/	3(15.8%)	7(36.8%)	9(47.4%)	<0.05
LED (mg/day)	/	390.02(204.672)	698(508.704)	689.34(339.458)	<0.01

Data are mean (SD) or n (%). P values correspond to three-group comparisons.

Abbreviations: HC, healthy control; PDD, PD-NC, Parkinson’s disease with no cognitive impairment; PD-MCI, Parkinson’s disease with mild cognitive impairment; Parkinson’s disease with dementia; UPDRS, Unified Parkinson’s Disease Rating Scale. ESS, Epworth Sleepiness Scale. GDS, Geriatric Depression Scale. SSST 12, the Sniffin’ Sticks Screening 12 Test. RBDSQ, Rapid Eye Movement Sleep Behavior Disorder Screening Questionnaire. LED, Levodopa equivalent dose. #Off-state motor ratings according to the UPDRS (motor section).

*chi-square test.

**Kruskal Wallis test.

Unsurprisingly, the scores on the MMSE and specific cognitive assessments were dramatically different among the PDD, PD-MCI and PD-NC patients. The patients’ detailed cognitive profiles are shown in Table 2.

**Table 2:** Neuropsychological evaluation of patients with Parkinson’s disease.

**Cognitive test**	**PDD**	**PD-MCI**	**PD-NC**	**p Value**	**Post hoc significance**
**MMSE**	19.41(4.521)	26.16(2.522)	28.33(1.06)	<0.001	[M<N^c^] [D<N^c^] [D<M^c^]
**Attention and working memory**					
SDMT	15.92(14.705)	20.06(10.031)	35.51(12.213)	<0.001	[M<N^c^] [D<N^c^]
TMT-A (s)	150(98.555)	91.08(40.139)	58.42(17.882)	<0.001	[M<N^c^] [D<N^c^]
**Executive function**					
CWT-C time (s)	126.67(60.405)	99.03(32.425)	74.36(17.879)	<0.001	[M>N^b^] [D>N^c^]
CWT-C right	38.59(5.28)	44.42(4.486)	45.9(3.447)	<0.001	[D<M^c^] [D<N^c^]
TMT-B (s)	267(194.498)	215.27(77.326)	147.37(50.864)	<0.001	[M>N^c^] [D>N^a^]
**Language**					
BNT	17.62(5.987)	20.32(4.19)	24(3.356)	<0.001	[M<N^c^] [D<N^c^]
AFT	8.909(4.8786)	13.838(3.6325)	16.718(4.1482)	<0.001	[M<N^b^] [D<N^c^] [D<M^c^]
**Memory**					
AVLT-delay recall	1.11(1.41)	3.35(2.031)	5(2.271)	<0.001	[M<N^c^] [D<N^c^] [D<M^c^]
AVLT-T	10.909(8.1935)	18.162(7.7514)	27.179(8.0094)	<0.001	[M<N^c^] [D<N^c^] [D<M^c^]
CFT-delay recall	4.32(4.177)	10.66(8.429)	15.74(6.155)	<0.001	[M<N^a^] [D<N^c^][D<M^b^]
**Visuospatial function**					
CFT	17.86(12.69)	26.44(8.262)	33.69(11.619)	<0.001	[D<N^c^] [M<N^c^]
CDT	11.41(7.874)	16.97(6.016)	22.47(5.54)	<0.001	[M<N^c^] [D<N^c^] [D<M^b^]

Data are mean (SD). P values correspond to three-group comparisons.

Abbreviations: PDD, Parkinson’s disease with dementia; PD-MCI, Parkinson’s disease with mild cognitive impairment; PD-NC, Parkinson’s disease with no cognitive impairment; MMSE, Mini Mental State Examination; SDMT, Symbol Digit Modality Test; TMT, Trail Making Test; CWT, Stroop Color-Word Test; BNT, Boston Naming Test; AFT, Animal Fluency Test; AVLT, Auditory Verbal Learning Test; CFT, the Rey-Osterrieth Complex Figure Test; CFT, Clock Drawing Test; N, PD-NC; M, PD-MCI; D, PDD.

^a^ p < 0.05, ^b^ p < 0.01, ^c^ p < 0.001

### MiR-29s and cognitive impairment in PD patients

**Figure 1:**
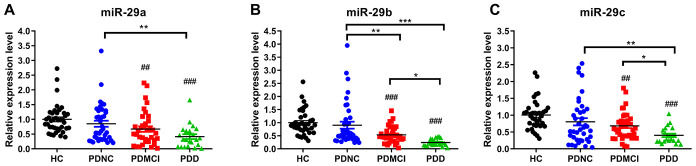
**Expression levels of miRNA-29s among PDD, PD-MCI, PD-NC groups and healthy controls.** (**A**) Expression levels of miRNA-29a among PDD, PD-MCI, PD-NC groups and HCs. (**B**) Expression levels of miRNA-29b among PDD, PD-MCI, PD-NC groups and HCs. (**C**) Expression levels of miRNA-29c among PDD, PD-MCI, PD-NC groups and HCs. Abbreviations: HC, healthy control; PDD, Parkinson’s disease with dementia; PD-MCI, Parkinson’s disease with mild cognitive impairment; PD-NC, Parkinson’s disease with no cognitive impairment. Pound sign shows the comparison with healthy control group. ##, p<0.01; ###, p<0.001. Asterisk shows the comparison within three PD groups. *, p<0.05; **, p<0.01; ***, p<0.001.

MiR-29a/b/c levels showed no differences between the PD-NC group and HC group (p>0.05). MiR-29a/b/c expression was significantly lower in the PDD group than those in the PD-NC group (Figure 1) (p<0.05). The expression levels of miR-29b and miR-29c were both significantly lower in the PDD group than those in the PD-MCI group (p<0.05). Additionally, miR-29b expression in the PD-MCI patients was significantly downregulated with respect to that in the PD-NC patients (p<0.01).

We built a univariate model (Model 1) to explore the “crude” effect of miR-29s on incident PDD or PD-CI. As shown in Table 3, the univariate logistic regression suggested significant associations between the expression of all members of the miR-29s family with PDD and PD-CI (PDD + PD-MCI). These associations remained significant after adjusting for years of education and UPDRS-III subscore in a multivariate model (Model 2) with the exception that miR-29c levels were not associated with PD-CI.

**Table 3:** Regression analyses of the association between cognitive impairment in patients with Parkinson’s disease and serum miRNA-29s.

	**PDD *vs.* non-PDD**			**PD-CI *vs.* PD-NC**		
	**β**	**S.E.**	**p**	**β**	**S.E.**	**p**
**Model 1**						
miRNA-29a	-1.391	0.580	0.017*	-0.718	0.329	0.029*
miRNA-29b	-7.310	1.873	<0.001***	-1.773	0.564	0.002**
miRNA-29c	-1.752	0.677	0.010*	-0.668	0.332	0.044*
**Model 2**						
miRNA-29a	-2.348	0.834	0.005**	-0.951	0.438	0.030*
miRNA-29b	-8.533	2.252	<0.001***	-1.901	0.745	0.011*
miRNA-29c	-2.510	0.902	0.005**	-0.833	0.436	0.056

Model 1: Univariate analysis; Model 2: Multivariate Logistic regression model, adjusted for years of education, and UPDRS-III subscore.

Abbreviations: PDD, Parkinson’s disease with dementia; non-PDD, Parkinson’s disease without dementia, the combination of PD-MCI and PD-NC; PD-CI, Parkinson’s disease with cognitive impairment, the combination of PDD and PD-MCI; PD-NC, Parkinson’s disease with normal cognition. * p<0.05; **, p<0.01; *** p<0.001.

**Figure 2:**
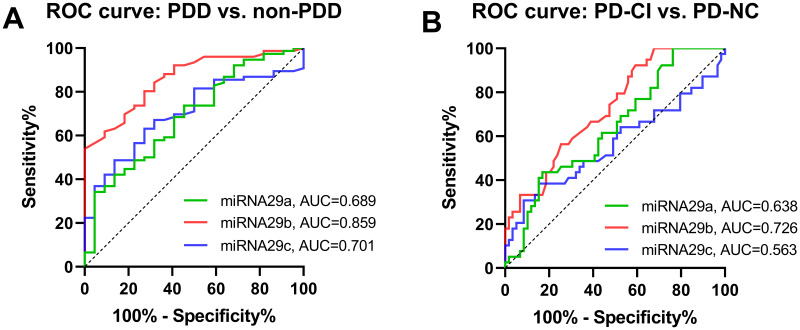
**Diagnostic utility of miRAN-29s for PDD and PD-CI.** (**A**). ROC curve for serum miRNA-29s differentially expression between PDD and non-PDD. (**B**) ROC curve for serum miRNA-29s differentially expression between PD-CI and PD-NC. The true positive rate (sensitivity %) is plotted as a function of the false positive rate (100 % - specificity %). Abbreviations: PDD, Parkinson’s disease with dementia; non-PDD, Parkinson’s disease without dementia, the combination of PD-MCI and PD-NC; PD-CI, Parkinson’s disease with cognitive impairment, the combination of PDD and PD-MCI; PD-NC, Parkinson’s disease with normal cognition.

ROC analysis was conducted to determine the diagnostic accuracy of miR-29s in distinguishing PDD from non-PDD (PD-MCI + PD-NC) and PD-CI (PDD + PD-MCI) from PD-NC. For discriminating between PDD and non-PDD, the area under the curve (AUC) of miR-29b, which indicates the accuracy, was considered high at 0.86 (0.859, 95% CI=0.7817 to 0.9372) (Figure 2A), while the AUCs of miR-29a and miR-29c were moderate (0.689, 95% CI=0.5655 to 0.8119 and 0.701, 95% CI =0.5917 to 0.8109, respectively). For discriminating between PD-CI and PD-NC, the AUC of miR-29b was moderate, with an average AUC of 0.726 (95% CI=0.6268 to 0.8243) (Figure 2B). Less impressively, miR-29a achieved an AUC of 0.638 (95% CI= 0.5276 to 0.7483), and miR-29c achieved an AUC of 0.563 (95% CI= 0.4390 to 0.6866).

### MiR-29s and cognitive domains in PD patients

After controlling for years of education and UPDRS-III subscore in the multivariate linear regression model, miR-29b expression and miR-29c expression were associated with the global cognitive status evaluated by the MMSE (β = 1.240, 95% CI=0.154 to 2.327 and β = 1.391, 95% CI=0.238 to 2.545, respectively). For specific cognitive domains, all miR-29s levels were associated with the z-score of memory function (β=0.455, 95% CI=0.090 to 0.821, β=0.453, 95% CI=0.096 to 0.81 and β=0.483, 95% CI=0.102 to 0.864 for miR-29a, miR-29b and miR-29c, respectively) in the multivariate linear regression model (Table 4). Moreover, miR-29a and miR-29b levels were associated with the z-score of language function (β=0.354, 95% CI=0.039 to 0.669 and β=0.339, 95% CI=0.033 to 0.645, respectively), and miR-29b level was associated with the z-score of executive function (β=0.309, 95% CI=0.060 to 0.559).

**Table 4:** Multivariate linear model results for association between miRNA-29s and cognitive domain z-scores of patients with Parkinson’s disease.

**Cognitive domain z-score**		**miRNA-29a**	**miRNA-29b**	**miRNA-29c**
		β=1.104	β=1.240	β=1.391
**Global**		95% CI=--0.025 to 2.233	95% CI=0.154 to 2.327	95% CI=0.238 to 2.545
		p=0.055	p=0.026*	p=0.019*
		β=0.181	β=0.198	β=0.257
**Attention**		95% CI=-0.092 to 0.454	95% CI=-0.053 to 0.449	95% CI=-0.019 to 0.532
		p=0.191	p0.121	p=0.068
		β=0.132	β=0.309	β=0.146
**Executive**		95% CI=-0.142 to 0.405	95% CI=0.060 to 0.559	95% CI=-0.140 to 0.432
		p=0.341	p=0.016*	p=0.312
		β=0.354	β=0.339	β=0.247
**Language**		95% CI=0.039 to0.669	95% CI=0.033 to 0.645	95% CI=-0.083 to 0.577
		p=0.028*	p=0.030*	p=0.140
		β=0.455	β=0.453	β=0.483
**Memory**		95% CI=0.090 to 0.821	95% CI=0.096 to 0.811	95% CI=0.102 to 0.864
		p=0.015*	p=0.014*	p=0.014*
		β=0.350	β=0.284	β=0.157
**Visuospatial**		95% CI=-0.198 to 0.898	95% CI=-0.251 to 0.818	95% CI=-0.417 to 0.730
		p=0.207	p=0.294	p=0.588

Multivariate linear regression model, adjusted for years of education and UPDRS-III subscore.

Cognitive domain z-scores were derived from two or more neuropsychological test scores.

*, p<0.05

### MiR-29s and clinical characteristics of PD patients

MiR-29s levels showed no correlation with the clinical parameters of interest, including age, gender, years of education, disease duration, LED, H&Y stage, UPDRS-III subscore, ESS score, GDS score, SSST-12 score, RBDSQ score or the incidence of hallucination (Table 5).

**Table 5:** Correlations between miRNA-29s and clinical characteristics of patients with Parkinson’s disease.

	**miRNA-29a^##^**		**miRNA-29b^##^**		**miRNA-29c**	
	**r**	**p value**	**r**	**p value**	**r**	**p value**
Age, years	0.117	0.251	0.122	0.233	0.114	0.262
Gender^##^	-0.059	0.567	-0.084	0.411	-0.008	0.940
Education, years	0.000	0.998	-0.003	0.975	-0.031	0.768
Disease duration, months^## ^	0.047	0.664	-0.113	0.292	0.049	0.648
UPDRS-III subscore	-0.097	0.350	-0.087	0.403	0.024	0.817
Hoehn and Yahr stage^##^	-0.029	0.776	-0.023	0.828	0.111	0.280
ESS score^##^	0.094	0.364	0.003	0.975	0.109	0.292
GDS score	-0.124	0.234	-0070	0.502	-0.039	0.707
SSST 12 score	-0.025	0.811	0.027	0.798	-0.015	0.882
RBDSQ score	0.058	0.587	0.031	0.768	0.077	0.468
Hallucination^##^	-0.098	0.338	0.016	0.878	-0.080	0.437
LED (mg/day)^##^	-0.043	0.713	-0.109	0.344	0.195	0.090

P values correspond to correlation between miRNA-29s and clinical characteristics.

Abbreviations: UPDRS, Unified Parkinson’s Disease Rating Scale. ESS, Epworth Sleepiness Scale. GDS, Geriatric Depression Scale. SSST 12, the Sniffin’ Sticks Screening 12 Test. RBDSQ, Rapid Eye Movement Sleep Behavior Disorder Screening Questionnaire. LED, Levodopa equivalent dose.

#Off-state motor ratings according to the UPDRS (motor section).

^##^ P values correspond to spearman correlation test for unnormal-distributed data.

## DISCUSSION

The present study compared serum miR-29a/b/c levels in PDD, PD-MCI, PD-NC patients and healthy controls. Low expression of miR-29s in PD patients was significantly associated with poorer cognitive function. In particular, miR-29b differentiated PDD from non-PDD with high accuracy and PD-CI from PD-NC with moderate accuracy.

Biomarkers that allow early identification of cognitive dysfunction have the potential to assist in predicting the onset of dementia in PD and could possibly lead to earlier interventions. Previous research has made efforts to identify biomarkers of PD-CI, including attempts to show relationships with the levels of Aβ in CSF, uric acid in plasma/serum, measurements of cerebral cholinergic innervation and metabolism using PET, and hippocampal size measured by MRI [15]. Either the invasive nature or high costs of these techniques preclude their routine use in large populations. This study is an attempt to widen the list of potential biomarkers to include blood-based miRNAs. Although many miRNAs have been identified to be associated with PD [16], to the best of our knowledge, this is the first study to directly explore whether miRNAs in blood could be peripheral biomarkers for cognitive impairment in PD.

miRNAs have been reported as peripheral biomarkers of other neurocognitive disorders, such as Alzheimer’s disease (AD) [5], vascular dementia [6] and frontotemporal dementia (FTD) [7]. Upregulation of miR-29c was shown to promote learning and memory behaviors in AD mice [17]. In this study, we illustrated downregulation patterns of serum miR-29s in PD with cognitive impairment. Specifically, the expression levels of miR-29s, particularly miR-29b, decreased in the PDD and PD-MCI groups compared with those in the PD-NC group. Furthermore, miR-29b, while having a high AUC in discriminating PDD from non-PDD and a moderate AUC in discriminating PD-CI from PD-NC, showed no correlation with other clinical parameters. After adjusting for other confounders, miR-29b continued to be associated with global cognitive parameters. The specific association with cognitive impairment makes miR-29b a potential candidate biomarker for distinguishing PDD patients.

In specific domains, we noted that the levels of the miR-29s family were all associated with the z-score of memory function. As PD-NC progresses to PDD, studies have shown that memory deficits become apparent, implicating a supervening dysfunction of temporal lobe storage mechanisms [18]. This association between miR-29s and memory function may result from the high expression of miR-29s in the central nervous system, particularly in neurons of the hippocampus [14]. Additionally, miR-29a and miR-29b showed an association with the language z-score, and miR-29b expression contributed to executive deficits, but the underlying mechanisms need to be further explored.

Our previous study showed a marked reduction in serum miR-29s levels in PD patients compared with those in the unaffected controls. In this study, we further divided PD patients into three groups according to cognitive status. However, serum miR-29s levels in the PD-NC patients and healthy controls showed no significant differences, but serum miR-29s levels in the PD-MCI and PDD patients were significantly lower than those in healthy controls. This might indicate that miR-29s are more related to cognition but not pure Parkinsonism. These results may be due to the different neurobiological bases underlying cognitive and motor deficits in PD. Additionally, our previous study found that serum miR-29s did not differ between AD patients and healthy participants [10]. It seems that serum miR-29s are associated with PD-CI but not AD.

Currently, the mechanisms underlying cognitive impairment in PD remain unclear [19]. miR-29s may provide insights into these pathological mechanisms. The target genes for mature miR-29a/b/c heavily overlap according to computer prediction. The synaptic regulator PARK7 (DJ-1), mitogen-activated proteins MAPK6, MAPK7, and MAPK10, short-term memory to long-term memory conversion regulator CREB, and neurogrowth and neurotrophic factors IGF1 and IGF2 are candidate targets of miR-29s. The pathogenetic role of miR-29s in PDD or PD-MCI warrants further study.

Our study has some limitations. First, this study has a small sample size. Thus, a larger group of patients is needed to confirm these results. Second, this study utilizes a cross-sectional design, which could not be used to analyze the longitudinal impacts of miR-29s on cognitive function in PD. Longitudinal studies are required to explore whether miR-29s could identify PD patients at risk for further cognitive decline. Additionally, our cross-sectional study could not make causal inferences, and the role of miR-29s in the pathogenesis of cognitive impairment in PD is worthy of further study.

In conclusion, we identified the association of the serum miR-29 family with cognitive impairment in PD. Our findings reveal that the serum microRNA-29 family, especially miR-29b, may be potential biomarkers for PDD and PD-CI.

## MATERIALS AND METHODS

### Participants

Between March 2012 and September 2018, 98 PD patients aged 50-80 years who agreed to participate were recruited from the Department of Neurology, Huashan Hospital. PD diagnosis for each participant was determined by two senior specialists of movement disorders according to the UK Brain Bank criteria [20]. Patients with any history of stroke, epilepsy, encephalitis, traumatic brain injury, malignancies, cardiac events, or severe psychiatric illness were excluded from the study.

Forty age- and gender-matched control subjects were voluntarily recruited. None of the control subjects had a history of neurologic/psychiatric disorders.

### Clinical evaluation

All participants went through clinical assessment after at least 12 h off anti-parkinsonian medications. Motor symptom evaluation included the Unified Parkinson’s Disease Rating Scale Part III (UPDRS-III) and the modified Hoehn and Yahr staging (H&Y), while nonmotor symptoms were measured by the Epworth Sleepiness Scale (ESS), the Geriatric Depression Rating Scale (GDS), the Sniffin’ Sticks Screening 12 Test (SSST-12) [21], and the Rapid Eye Movement Sleep Behaviour Disorder Screening Questionnaire (RBDSQ). Total daily levodopa equivalent doses (LEDs) were calculated to represent the doses of PD medications.

### Neuropsychological assessments

All participants underwent the Mini-Mental State Examination (MMSE) for global cognitive evaluation [22] and the following comprehensive neuropsychological battery for five specific cognitive domains: (1) attention and working memory: Symbol Digit Modality Test (SDMT) [23] and Trail Making Test A (TMT-A) [24]; (2) executive function: Stroop Color-Word Test (CWT) [25] and Trail Making Test B (TMT-B) [24]; (3) language: Boston Naming Test (BNT) and Animal Fluency Test (AFT) [26]; (4) memory: Auditory Verbal Learning Test (AVLT) [27] and delayed recall of the Rey-Osterrieth Complex Figure Test [28]; (5) visuospatial function: Clock Drawing Test [29] and the copy task of the Rey-Osterrieth Complex Figure Test [28].

All participants were in the ON condition during cognitive assessment to minimize the confounding impact of motor symptoms. To obtain normative data for the Chinese adult population, we recruited 100 healthy controls matched for age, education, and gender (Supplementary Table 1). The raw score of the individual neuropsychological tests was transformed into a z-score by subtracting the mean test score of the control sample from the individual raw scores and then dividing the difference by the standard deviation of the score of the control sample according to the following formula:

$$\text{Z}\;\text{score}=(\text{test}\;\text{score}-{\text{Mean}}_{\text{control}}){\text{/SD}}_{\text{control}}.$$

The mean of two z-scores for each domain was used as the compound score.

PDD was diagnosed by the current clinical diagnostic criteria of the Movement Disorder Society (MDS) Task Force 2007 [30, 31]. PD-MCI was defined based on the MDS Task Force 2012 (Level 2) criteria [32], and impairment (>1.5 SD below the normative mean) on at least 2 neuropsychological tests within the same cognitive domain or across different domains was required. PD-CI includes both PDD and PD-MCI. Non-PDD includes both PD-MCI and PD-NC.

### Reverse-transcription quantitative real-time PCR (RT-qPCR) for miRNA

Serum samples were collected from each participant on the same day as the clinical and neuropsychological assessments. Serum miRNAs were extracted using a miRNeasy Serum/Plasma Kit (Qiagen, Germany), during which proportional miRNeasy Serum Spike-In Control was added as the reference RNA. Reverse transcription was performed with a miRcute miRNA First-Strand cDNA Synthesis Kit (Tiangen, China), followed by quantitative real-time PCR of 2 μl of the product. The PCR primer sequences (purchased from Life Technologies) were as follows: miR-29a (5’-TAGCACCATCTGAAATCGG-3’); miR-29b (5’-TAGCACCATTTGAAATCAGT-3’); and miR-29c (5’-TAGCACCATTTGAAATCGG-3’). Relative expression levels were calculated using the 2-ΔΔCt method and normalized to the level of the reference RNA. The whole RT-qPCR process was assessed by two experienced researchers blinded to the clinical and neuropsychological data.

### Data analysis

Continuous variables are shown as the means ± SDs, while categorical data are presented as numbers and frequencies (%). Data were analyzed using IBM SPSS Statistics (version 21). P < 0.05 was defined as statistically significant.

For quantitative data, differences among the three PD groups were compared by one-way analysis of variance (ANOVA) with an LSD post hoc test and the Kruskal-Wallis test for normally distributed and nonnormally distributed data, respectively. Categorical data were analyzed with the chi-squared test or Fisher's exact test as appropriate. To test the diagnostic accuracy of miR-29s, we used receiver operating characteristic curve (ROC) analyses.

We calculated correlations between miR-29s expression levels and clinical parameters (age, years of education, H&Y, UPDRS-III, etc.) using Pearson’s (r) correlation or Spearman’s (rho) correlation as appropriate. A univariate model and a multivariate model were used to explore the effect of miR-29s expression levels on incident PDD or PD-CI with or without considering other confounders (including years of education and UPDRS-III subscore). Controlling for the same covariates, independent associations of miR-29b expression levels with cognitive domain z-scores were evaluated by multivariate linear regression analysis.

### Ethics statement

This investigation was conducted in accordance with the ethical standards of the Declaration of Helsinki and according to national and international guidelines, and has been approved by the Human Studies Institutional Review Board, Huashan Hospital, Fudan University. All participants provided written informed consent.

## Supplementary Material

Supplementary Table 1
