# Supplementary material for: Association of the serum microRNA-29 family with cognitive impairment in Parkinson's disease
Source: Aging (Albany NY). 2020 Jul 9;12(13):13518–28. doi: 10.18632/aging.103458 (PMC7377865; doi:10.18632/aging.103458)
Supplement: Supplementary Table 1 [file aging-12-103458-s001..pdf]

SUPPLEMENTARY TABLE

Supplementary Table 1. Cognitive profile of healthy controls.

Cognitive test	Number(n)	Mean	SD
**MMSE**	93.00	27.85	1.87
**Attention and working memory**			
SDMT	97.00	34.78	15.20
TMT-A (s)	97.00	48.71	28.24
**Executive function**			
CWT-C time (s)	99.00	77.87	21.00
CWT-C right	99.00	46.80	3.54
TMT-B (s)	97.00	121.37	64.24
**Language**			
BNT	100.00	24.82	3.22
AFT	97.00	18.02	4.35
**Memory**			
AVLT-delay recall	100.00	5.93	1.41
AVLT-T	100.00	29.54	6.21
CFT-delay recall	100.00	16.44	5.52
**Visuospatial function**			
CFT	100.00	33.51	3.25
CDT	100.00	25.81	6.31

Abbreviations: MMSE, Mini Mental State Examination; SDMT, Symbol Digit Modality Test; TMT, Trail Making Test; CWT, Stroop Color-Word Test; BNT, Boston Naming Test; AFT, Animal Fluency Test; AVLT, Auditory Verbal Learning Test; CFT, the Rey-Osterrieth Complex Figure Test; CFT, Clock Drawing Test.
